# Mortality Statistics of the U.S. Census for 1850

**Published:** 1858-03

**Authors:** Ezra Read

**Affiliations:** Terre Haute, Indiana


ART. VIII.—*The Mortality Statistics of the Census of* 1850, *and a Review of the Letter of Dr. Edward Jarvis upon the Classification of Diseases.* By EZRA READ, M.D., of Terre Haute, Indiana.

IN the absence of a general registration law, the Government of the United States, in taking the census of 1850, made an effort, for the first time, to collect all the deaths, with their causes, which had occurred the previous year. Imperfect as it must have been, it was yet a matter of the greatest importance, and was but an earnest or foreshadowing of what may hereafter take place. It was fairly presumed that each family would have a distinct remembrance for a year, at least, of an event so impressive to every household. Most families, however, do not rely upon their memories, but keep a family record as a matter of reference.

Where no member of the family can write, some friend is found who performs this pious duty from time to time, as required, and the record is religiously preserved as a family history, and is recognized in courts of law as truthful. It is a prevailing custom, and so general that, in the absence of a registration law, this of itself, if a transcript were taken from each family, would form a very correct history of the births, marriages, and deaths of the country. Most families, too, are careful to ascertain the name of the disease of which its member died. Where medical attention was had, it may be presumed that there was sufficient precision in designating the cause of death. This, however, is not always to be relied upon, as the medical attendant may sometimes, from carelessness, give the name of the prominent closing symptoms, instead of the primary disease. As, for instance, a child dies in convulsions, caused by an acute disease. Here it may readily be said to have "died of convulsions," when, in reality, it may have been the result of diarrhœa, scarlatina, measles, small-pox, or any other acute affection.

Where there has been no medical attention, the accuracy in naming the disease cannot be regarded so perfect. Here, however, there is always some one found sufficiently skilled in diseases to give it a name. At all events, returns were made of the deaths under some name or head. It then became a matter of no small importance to reduce this crude matter to order, and to embody the various popular names under their scientific synonyms. It was especially due to the profession of medicine that this work should be done accurately, as it forms the embodiment of a mortality history for the middle of a century the most remarkable for progress of any in the history of the human race; and at a period when the science of medicine had attained a position greatly in advance of any that had ever preceded it. This record was made when the millions of our busy population were swaying to and fro under the mightiest impulses which ever controlled the human mind or ruled its actions. It was made when we rushed to the sea, and upon its surface, in multitudes, for the rich returns and gains of our prosperous commerce. It was made when the boundless prairies of our new States and Territories were dotted with human beings preparing new homes in a strange climate; and when the virgin forests were filled with the emigrant, surrounded by the pestilential exhalations of its shadowed surface. It was made when thousands of our citizens were engaged in constructing railroads in every State; when machinery was brought to its highest perfection; when the human mind was wrought to its highest tension in every pursuit that enriches, enlightens, or advances society. It was made when the highways of our country were thronged with a foreign population, just landed upon our shores. It was made when our gold-mines were inviting thousands of all ages and pursuits, by a long and perilous overland route, or by that which was still more fatal, the Panama route. It was made when great numbers of our population were submitted to almost entirely new physical conditions. It was made at an age when the body ran and the mind flew, when to rest was to retrograde. How vastly important then, to the character of this great age, that science should carefully indicate and truthfully preserve the names of the diseases which produced death among a people so variously located and so differently employed. It was due to other generations to transmit a faithful record of its diseases.

Every decade of years has its own peculiar diseases. Suddenly from those of a new country we have those of an old, so short is the time required, in this busy age, to change the new to the old. The comforts and luxuries attendant upon wealth bring with them a train of diseases unknown to those in penury and want, and *vice versâ.* Those of crowded cities differ from those of rural districts, and those of the industrious from the idle. Many others are provided against by the ingenuity of man. He drains the soil; he tills the earth; he surrounds himself with comforts which are protecting agents against disease. The pioneer encounters a class of ailments which yield to cultivation and improvement; hence it is that the diseases of new countries differ from those of the old. Hence, too, the necessity of transmitting to posterity an accurate and faithful account of our diseases, and the changes which are wrought from one decade to another, by habit and pursuit, by cultivation and drainage of the soil, by the introduction of new and improved machinery, by better ventilation, and by improved methods of heating public and private buildings, and, in fact, by all causes which induce a change in any way whatever.

To render this, the very beginning of our mortality statistics, as accurate as possible, Mr. De Bow, the Superintendent of the Census Bureau, invoked the aid of Dr. Edward Jarvis, of Massachusetts, to classify the variously named diseases which were found upon the marshals' returns. Notwithstanding this was a task of a good deal of labor and research, yet it might have been perfectly done, so far as the returns showed. Inasmuch as Dr. Jarvis, in many respects, failed to make a correct classification of diseases, and has placed them where they do not belong, and assigned to them erroneous names, I regard it as a duty I owe the profession of medicine to aid, as far as I can, in correcting some of his errors, and to see that the names of our diseases are truthfully classed and faithfully transmitted to those who may come after us. Dr. Jarvis was not responsible for any inaccuracies which were found upon the marshals' returns. He was not responsible for the proper or true signification of any terms which were too vague to admit of a faithful classification; but he was responsible for inaccuracies and erroneous classifications which he himself made, and which it was his duty to have avoided. He might have taken all the diseases of the returns which had not a specific signification, or which admitted of a doubt, and placed them under the head of *unknown.* All others could have been assigned to their distinct and known classes, and the popular names could have been translated into their scientific synonyms. Having done this his labors would have been faithfully executed. In his letter to Mr. De Bow, he states that he reduced the number of diseases from 148 to 114. Let us examine in what manner this was done.

"Chicken-pox and hives," he says, (*Mortality Statistics,* page 46,) "should be in diseases of the skin. Herpes, ringworm, and tetter are to be included in the same."

The three last being one disease, we then have three diseases—chicken-pox, hives, and herpes, having no other resemblance or analogy to each other than their general location upon the skin—placed together under one head, (diseases of the skin,) which conveys no more adequate idea of the cause of death than would the simple announcement, died of disease. It is not enough to declare that these diseases are not mortal, or that death was the result of some other disease. We are dealing with facts, and have no right to assume anything not expressed in the returns. It would have been more in accordance with the duty assigned him to have allowed these to remain just as returned, with a simple appended note stating that these diseases were not regarded mortal. The reader then would very properly have been permitted to draw his own inference. Again, he says:—

"Disease of the eyes *may* involve disease of the brain; but otherwise is not fatal. It should, therefore, be included in diseases of the brain and nervous system."

Upon this principle of reasoning, a very large class of diseases might be transferred to the same head. In fact, all which in their progress involve the brain and nervous system. In this respect he sustains his consistency, for immediately following he says:—

"Mumps comes under the same category as the last, (disease of the eyes.) It may be connected with disease of the brain, and it may coexist with other and fatal disease. It would be safer to put these cases in the *unknown.*"

He seems here to have trembled at his own temerity, when he mixed together such dissimilar diseases, and united the *sociabile* to the *dissociabili,* and therefore scratched mumps from diseases of the brain and nervous system, and placed it with the *unknown.*

"Onanism, possibly, but not probably, was a cause of death. It wastes life and produces other disorders. Cases under this head should be put under the *unknown.*

"Suppression of the menses (amenorrhœa) is a symptom or consequence of many other diseases. It gradually happens in diseases of high excitement, as fevers, etc.; and also in diseases of waste,—consumption, scrofula, and cancer, especially uterine. It may be safer to put these cases in the diseases of the organs of generation."

Without transferring his remarks in full, I would state that he places *fluor albus* in diseases of the organs of generation; vaccination in the *unknown;* amputation in the *unknown;* cachexia in the *unknown.*

It occurs to me that the profession of medicine will not sustain him in placing so much heterogeneous matter in the *unknown,* especially as the above-named diseases in their heading are clear and specific, conveying clear and specific ideas of what is meant.

"Congestion is equally unintelligible, and should be in the same class, the *unknown.*"

Whatever may be its signification elsewhere, in all malarious regions there is nothing unintelligible in the term *congestion.* Its definition is "Excess of blood in the vessels of a part, with diminished motion of that blood." Wherever, then, this local hyperœmia takes place in a vital organ to such an extent as to interrupt its functions, death supervenes. This is of frequent occurrence wherever periodical diseases prevail. Sudden deaths arise from a functional interruption in some one of the vital organs—the stomach, liver, brain, or lungs. It is *congestion,* and this term has a signification which conveys to the mind of any medical man a perfectly correct and full idea of the cause of death, although it may not specify the organ congested. No other term would convey it, and there can be no more intelligent or significant heading than Died of Congestion. It is a term everywhere used by physicians in writing and speaking, and is well understood. In "Williams' *Principles of Medicine,*" may be found a valuable section devoted to *congestion,* and yet we are told by Dr. Jarvis that it is "unintelligible."

Immediately following this he says:—"Congestion of the brain probably is apoplexy; yet it may not be. Other affections may have been intended. It should, then, be in diseases of the brain."

Apoplexy was never intended when congestion of the brain is used. Their signification is distinct, and they should have a separate head. The former has reference to those attacks where the patient falls down suddenly, deprived of sense and motion, and lies like one in a deep sleep. He is, in a moment, deprived of all volition, and is entirely insensible. It is a disease of all countries, and is rather consequent upon free living, and is most apt to seize those who have passed middle life and are of the apopletic habit; while the latter (congestion) usually supervenes upon a chill, accompanied with intense pain of the head, which increases, with an imperfect reaction, until insensibility comes and death supervenes. Death, from this cause, is most common in malarious countries. It may occur in all ages and sexes, while apoplexy is rare in malarious regions, and is confined, as before stated, almost entirely to those who have passed the middle of life, and is rarely found to attack females. When he, therefore, places this disease in "diseases of the brain and nerves," he fails to convey to the mind that specific and precise idea that congestion conveys. Apoplexy and congestion of the brain should not be placed under one head, but should remain separate and distinct. After speaking of cramp and eruption, he again says:—

"Brain, congestion of, probably intended for apoplexy. Yet it may be used for other cerebral affections which are not revealed in the returns. I have placed *this*, therefore, in diseases of brain and nerves."

He manifests a stronger determination to blot out the word congestion than to study its import and give it the place it merits.

"Fever, bilious, is a most vague and uncertain term. In some regions it seems to be used for all sorts of fevers, and the word *bilious* for all sorts of difficulties of the digestive organs.

"Gastric fever is probably intended for inflammation of the stomach. Yet it is in many places used as vaguely in respect to fever as the word bilious.

"Winter fever is more vague than either of the last two. It may mean bilious, or typhus, or synochus. These three, with specific names, convey no specific idea, and they should be included under the general name of *fevers*, without further description.

"Congestive fever is merely a form or phase of typhus.

"Inflammatory fever is a name sometimes given to cases of typhus. These two are, therefore, included in typhus fever."

The term bilious fever has a much less extended signification than might be inferred from the foregoing paragraph. It has not a vague and uncertain meaning, nor is it used for all sorts of fever, but is confined to the intermitting and remitting fevers of malarious districts. Nor is the word bilious used for "all sorts of difficulties of the digestive organs," but refers to those cases where the patient has a foul and loaded tongue, with nausea of stomach and bitter taste in the mouth. In this respect it is specific enough, and should be placed under the head of remitting or intermitting fever, which are but different degrees of the same fever.

Gastric fever is nowhere used in any other sense, I think, than to designate inflammation of the stomach.

Winter fever has not a vague signification, as the doctor represents, but has a complete and exact meaning. It is the popular term for pneumonia, and never means anything else. Nor does it ever, as he states, mear bilious, or typhus, or synochus. No term in medicine has a more uniform and exact meaning. He places, however, bilious fever and gastric fever under one general head, that of *fevers.* I can scarcely conceive a greater error embodied in a smaller space than when he unites congestive and inflammatory fever, and places them under the head of typhus. They are separate and distinct in all respects; they are cognate in none. The one is a disease of summer and autumn, the other of the winter and spring. By what means can he make them one? Typhus fever would be greatly swelled beyond its true bounds, because all of the typhus, congestive, and inflammatory fevers are placed under typhus. The general name of *fever* would also be greatly extended, because *bilious, gastric,* and *winter fevers* are placed under it. Pneumonia would be lessened greatly below what really exists, because winter fever, which is its popular name, is placed under a different head. Most of the above are not convertible terms, nor are they synonyms. They should, therefore, be preserved distinct.

"Milk leg, not in itself fatal, yet was an accompaniment of the fatal disease. I include this in child-birth."

Milk leg, or its synonym, phlegmasia dolens, expresses just what we want, while child-birth may express many conditions; therefore the former should not be included in the latter.

He places canker, canker rash, and putrid sore throat under the head of scarlatina. The two first are rightly classed. The latter, I presume, is intended to express erysipelas, or black tongue. In the West putrid sore throat is so used. He includes diseases of the head in those of the brain. Inflammation of the brain in cephalitis. Inflammation of the lungs in pneumonia. Inflammation of the throat in laryngitis. Killed is included in accident, and lockjaw in tetanus. Some other diseases are then enumerated, which are very properly classified.

Wherein the returns of the cause of death were made under a scientific name, in no event should that have been changed or another substituted for it. Such a license would invalidate the truthfulness of the returns, and would render them unworthy the title of a record of the causes of mortality. Where popular names are used, it was obviously proper to use in their stead the scientific; yet, as a matter of history, the popular names should have had a separate place in the statistics, with their signification attached.

It is a matter of so much importance that popular names of diseases should be faithfully translated,—a matter of so much importance now and hereafter, that our mortality statistics should convey an accurate and exact idea of our diseases, that I would suggest to Government the propriety of forming a commission of physicians, one from each State, to superintend this department of the next census of the United States.

